# The role of human thermoregulation in thermal discomfort in lower-limb prosthetics: A scoping review

**DOI:** 10.33137/cpoj.v8i1.43073

**Published:** 2025-03-28

**Authors:** R Edwards, L Murray, A Buis

**Affiliations:** 1 Department of Biomedical Engineering, Faculty of Engineering, University of Strathclyde, Glasgow, Scotland.

**Keywords:** Literature Review, Scoping Review, Thermal Discomfort, Lower Limb Amputation, Prosthesis, Skin, Prosthetic, Amputation, Body/Device Interface, Thermoregulation, Temperature, Perspiration

## Abstract

**BACKGROUND:**

Thermal discomfort is one of the most prevalent issues experienced by lower-limb prosthetic users where, on average, 54% of users report thermal-related issues. This arises from wearing a prosthetic socket, which may disrupt the thermoregulatory system due to the low thermal conductivity of materials used in prosthetic sockets and liners. Despite the reported prevalence, there is little understanding of the impact of wearing a prosthesis on the body’s thermoregulatory system and how users perceive thermal discomfort.

**OBJECTIVE(S):**

This review aimed to evaluate the current understanding of how human thermoregulation correlates with subjective measures of thermal discomfort among lower-limb prosthetic users. It sought to gain a deeper understanding of how thermoregulatory parameters compare and relate to the subjective experience of thermal discomfort in this population.

**METHODOLOGY:**

The study design followed a scoping review structure to identify gaps in knowledge on the topic. A literature search was conducted across five online databases: Medline (ProQuest), EMBASE, Cochrane, CINAHL and PsycINFO. The searches covered literature from the earliest available date in each database up until February 2024. A search strategy was created to identify the relevant literature. An inclusion/exclusion criterion was then applied to identify studies that only measured either physiological or psychological aspects of thermoregulation and compared these aspects to thermal discomfort/comfort feedback. The QualSyst critical appraisal tool was used to gain quality score for each included article.

**FINDINGS:**

8 articles were identified for inclusion in this review, confirming a dearth in research into how wearing a prosthesis affects thermoregulation at the body/device interface (BDI) and the perception of thermal discomfort. Furthermore, it raised question to the relevance of using residual limb skin temperature measurements to assess thermal discomfort in lower-limb prosthetics. Perspiration at the BDI emerged as a potentially significant contributor to thermal discomfort, a consensus reflected in the literature.

**CONCLUSION:**

Despite significant technological advancements, thermal discomfort remains a persistent issue. Therefore, further research is warranted to further understand how wearing a prosthesis affects the thermoregulatory system, enabling the development of innovative components which can mitigate thermal discomfort and in turn improve the quality of life of lower-limb prosthetic users.

## INTRODUCTION

Acquired amputation can result from a number of pathologies such as diabetes, vascular disease, cancer and trauma. Globally, in 2017, 58 million people were living with an amputation due to traumatic causes.^1^ At a regional level, between 1990 and 2019, South Asia saw the highest prevalence during this period with 100 million traumatic amputations.

Traumatic amputation accounts for 45% of all amputations. Individuals in this group typically have no additional comorbidities or pathologies and are likely to return to a level of activity similar to their pre-amputation status.^2^ However, these prosthetic users who are wearing the prosthesis for longer periods, and actively engaged in employment and hobbies, often report complications related to thermal discomfort, regardless of geographical location.^3^

The scientific literature regarding thermal discomfort and perspiration has been documented by multiple authors. It is for instance reported, that persons who wear a prosthesis are more susceptible to increased heat and perspiration, evident in the fact that, on average, 54% of prosthetic users report thermal-related issues.^3^ Klute et al. described that susceptibility arises from wearing a prosthetic socket, which may disrupt the thermoregulatory system due to the low thermal conductivity of the materials used in manufacturing prosthetic sockets and liners.^4^ Despite the reported prevalence of the issue, there is little understanding of the impact of wearing a prosthesis on the body's thermoregulatory system and how users perceive thermal discomfort. Current research into thermal discomfort has been focused more on lower-limb prosthetics with one study reporting that people with a transfemoral amputation rated thermal discomfort to be higher compared to people with a transtibial amputation.^5^

Overall prosthetic socket comfort is essential for prosthetic use and, clinicians and prosthetic companies strive to deliver the best fitting and most comfortable sockets possible. This is to ensure user satisfaction, a reduction in prosthetic abandonment and increasing quality of life.^6^ Ensuring a good socket fit requires appropriate force transmission coupled with a reliable suspension method provided by a bespoke, socket. This, however, encloses the residuum which can hinder heat exchange, leading to elevated skin temperature and perspiration at the body/device interface (BDI). In addition, patella tendonbearing sockets are frequently being replaced by total surface-bearing sockets, which often require an elastomeric liner which, although functionally advantageous, could increase the incidence of prosthetic-related thermal stress.^7^

The skin is vital for thermoregulation, enabling heat exchange between the body and the external environment, known as sensible heat transfer. This process involves heat loss through subcutaneous blood vessels via vasodilation, evaporative heat loss via sweating and the flattening of hairs to remove the insulating layer of air over the skin.^8^ Donning a prosthesis impairs heat transfer between the residual limb and the environment for all three of these mechanisms by creating an impermeable barrier at the BDI. This creates the potential for an unnaturally warm and moist environment, leading to skin pathologies and functional issues with the prosthesis. Users may experience skin irritation or blistering which combined with the unfavorable environment can lead to infections.^9^

Pistoning, known as relative motion between the residual limb and the socket, can also occur due to micro-film lubrication as a result of sweat build-up, affecting socket suspension. Henao et al. discovered an increase in the coefficient of friction at the BDI in the presence of sweat which, when coupled with pistoning, could rapidly increase the onset of blistering.^10^

The human thermoregulatory process tightly regulates body core temperature around 37°C against thermal disturbances to maintain homeostasis and can be divided into two categories: autonomic and behavioral. The autonomic thermoregulatory process is involuntary and can alter heat production and dissipation through shivering or non-shivering thermogenesis, or as aforementioned, through vasodilation of subcutaneous blood vessels and evaporative heat loss.^11^ Behavioral thermoregulation is a voluntary process whereby a conscious decision is made to adapt to a change in the thermal environment. For example, seeking shade in a hot climate or adding clothing when too cold.^12^ Human perception of thermal discomfort may play an important role in activating behavioral thermoregulation. Discomfort arises when thermal stimuli result in a deviation of core temperature from the norm.^13^

Hensen et al. has defined thermal comfort as “*that condition of mind which expresses satisfaction with the thermal environment*”.^14^ It was also defined by The American Society of Heating, Refrigerating and Air-Conditioning Engineers (AHSREA) as “*the condition of mind in which satisfaction is expressed with the thermal environment*”.^15^ These definitions allude to the fact that thermal comfort is a state of mind, rather than a state condition, indicating its subjectivity with it being influenced by personal differences in mood, culture and other individual, organizational and social factors.^16^ While thermal discomfort plays a role in autonomic thermoregulation, it primarily drives behavioral thermoregulation due to its significant influence on skin temperature. Skin surface temperature has a relatively greater contribution to subjective thermal discomfort than the autonomic response. Due to this, thermal comfort initiates behavioral thermoregulation before autonomic thermoregulation. This is due to autonomic thermoregulation being a more metabolically demanding response that maintains body temperature.^17^ When wearing a prosthesis, the heat exchange processes through autonomic thermoregulation could be compromised. In addition, the behavioral thermoregulatory act is for the user to remove their prosthesis. This should not have to occur, but if necessary, this may not always be possible during day-to-day activities, compromising this thermoregulatory process.

With the knowledge that thermal discomfort may play a key role in both autonomic and behavioral thermoregulation, and that numerous prosthetics users report thermal discomfort, this scoping review has studied the available literature that compares thermoregulation to thermal discomfort in lower-limb prosthetics. It also assessed the breadth and depth of understanding of the impact that wearing a prosthesis has on the fundamental physiological processes involved in thermoregulation at the BDI.

## METHODOLOGY

### Search Strategy

A scoping review was undertaken to assess the current knowledge relating to the involvement of human thermoregulation in thermal discomfort for lower-limb prosthetic users. This review aimed to assess the different thermoregulatory factors, either physiological or psychological, to gain a deeper understanding of how human temperature regulation may be influencing thermal discomfort or vice versa. The methodology for this literature review was chosen to identify research gaps to guide future experimental research into the perception of thermal discomfort in lower-limb prosthetics.

A literature search was conducted between 15/01/24 and 02/02/24. The search was completed across 5 online databases; Medline (ProQuest), EMBASE, Cochrane, CINAHL and PsycINFO and the results were transferred to EndNote. Keywords were combined with Boolean operators to create a search strategy. The strategy utilized is highlighted in **Table 1**.

**Table 1:** Search strategy used across all five databases.

	Search terms
1	Thermoregulation OR “Thermal response” OR Temperature
2	Prosthe^*^ OR “Artificial Limb” (MeSH)
3	“Residual Limb” OR Stump
4	Amputee (MeSH) OR Amputa^*^
5	1 AND 2
6	1 AND 3
7	1 AND 4

### Search Selection

The searches spanned the period from the earliest date of each database until February 2024. All chosen articles from each database were then transferred into the EndNote (Version 20.3.0.17787) reference management software, and all duplicates were removed. Search results from each database were screened by title and abstract. The relevant articles which matched the inclusion and exclusion criteria, as presented in **Table 2**, were then chosen. Articles were included if the abstracts discussed or measured thermoregulation and/or thermoregulatory processes in either physiological or psychological terms and made a comparison to thermal discomfort in lower-limb prosthetics. Articles inclusion was assessed by two researchers. Four articles were identified by one reviewer and not the other and were discussed as a group.^18–21^ Upon discussion, these articles were not included in the review. Although they measured thermoregulatory parameters and addressed thermal discomfort as an issue, they did not discuss or record subjective measurements for comparison with the thermoregulatory parameters.

**Table 2:** Inclusion and exclusion criteria.

Inclusion criteria	Exclusion criteria
Studies published in English. Only peer-reviewed. Studies that measured thermoregulation and/or thermoregulatory processes and made a comparison to thermal discomfort feedback. Studies that included lower-limb prosthetics users.	Literature review studies. Studies that did not measure physiological or psychological aspects of thermoregulation and make a comparison to thermal dis/comfort feedback. Studies conducted on animals or prototypes.

### Data extraction

Data from the remaining articles were extracted and documented in a data extraction table (**Table 3**), providing an overview of the current literature and highlighting emerging themes. The information gathered was as follows: author, year of publication, location of the study, sample size, study design, participant demographic, testing interventions temperature/perspiration outcomes and the QualSyst quality threshold score.^22^ The findings are summarized in the attached data extraction table.

**Table 3:** Data extraction table.

Author; year; location; sample size	Study design	Participant demographic: amputation; age	Measurement tools utilised	Temperature/perspiration outcomes	QualSyst Quality Threshold Score
Diment *et al.* 2019; Oxford;30^5^	Comparative study.	TF 11, TT 19; 23–65yr	Clinical infrared thermometer. 6-point heat and sweat discomfort scale.	**Primary: i).** Skin temperature change. Findings: ANOVA test showed that on average, the skin on both legs cooled during exercise (0.085°C/min, *p* = 0.002). There was no significant difference in skin temperature between the amputated and contralateral limbs. A significant difference in skin temperature was observed between the front and back of the leg with the back being significantly warmer post-exercise. **Secondary: i).** Variation in temperature response between amputated and contralateral limb. Findings: Temperature changes were small, with a large standard deviation of approximately 0.5°C on each limb. The average deviation between the 3 measurements taken at each location was 0.021°C.	0.86
Gnyawali *et al.* 2023; Indianapolis; 9^28^	Randomised controlled trial.	TF 46.5yr ± 14.03	Vented linersocket system (VS, Ossur). Non-vented socket (nVS, Ossur). Data logger system with humidity and temperature sensors. 10-point perceived sweat scale. CLASS Survey	**Primary: i).** Skin humidity change. Findings: No meaningful changes were observed for both sound and a residual limb during baseline testing. Humidity significantly increased during activity compared to the baseline on the residual limb compared to the sound side. The relative residual limb humidity was significantly lower during activity with the use of the VS compared to the nVS. **ii).** In-socket/skin temperature change. Findings: No significant increase between the (nVS vs VS). There was also no significant difference in skin temperature between the residual and sound limbs. **Secondary: i).** Perceived sweat score Findings: Significantly lower in the VS group compared to the nVS group.	0.83
Klute *et al.* 2016; Seattle;5^25^	Randomised control trial.	TT; 18–70yr	DAE socket. TSB suction socket. Two thermistor sensors. Gravimetry. Prosthesis evaluation questionnaire. Custom, selfreporting questionnaire.	**Primary: i).** Residual limb skin temperature change. Findings: No difference between the two prostheses was observed during the rest-walk-rest protocol. **ii).** Accumulated/expelled perspiration. Findings: The DAE prosthesis accumulated 1.09 ± 0.90g and expelled 0.67 ± 0.38g of perspiration. The Suction prosthesis accumulated 0.97 ± 0.75g of perspiration. **Secondary: i).** Residual limb skin temperature increased ~3°C for both prostheses during the 30-minute treadmill walk.	0.79
Hasegawa *et al. 2020;* Hiroshima; 14^30^	Randomised controlled trial.	TF 5, TT 2, C 7; LLA (36.4yr ± 4.4); C (31.7yr ± 7.8)	A rectal thermistor. Thermistor sensors. Body surface area DuBois formula. Sweat capsule and a local sweat meter. Laser Doppler blood flow meter.	**Primary: i).** Rectal temperature change. Findings: Increased throughout the test however no difference was observed between the groups. **ii).** Skin temperature change. Findings: Increased throughout the test in both groups however intended to be higher in the LLA group. **iii).** LSR change. Findings: Increased throughout the test for both groups at the thigh. A difference between the left and right leg was not observed in the C group. In the LLA group, LSR was significantly greater on the non-amputated side compared to the amputated side. The total sweat rate was also significantly higher in the LLA group.	0.73
Wernke *et al.* 2015; Ohio, 16^27^	Double-blind randomised, crossover design.	TT (1 BL); 32–78yr	SmartTemp liner and placebo liner. 4 thermocouples. Gravimetry.	**Primary: i).** Skin temperature change. Findings: The mean increase after activity was 0.2°C higher for the placebo liner compared to the SmartTemp liner. **ii).** Accumulated perspiration. Findings: Significantly reduced when wearing the SmartTemp liner for 12 participants post-activity **Secondary: i).** Inclusion of a bilateral amputee for testing both treatments in the same activity. Skin temperature increased during activity on both limbs, however, skin temperatures associated with the SmartTemp liner were lower compared to the placebo liner.	0.68
Segal *et al.* 2016; Seattle; 8^26^	Structured observational study	TT; 31-64yr	Four thermistor sensors. 11-point Likert psychometric scale. Ingestible temperature sensor. Polar heart rate monitor.	**Primary: i).** Residual limb skin temperature change. Findings: Mean temperature increased from 30.6 °C ± 2.6°C at Rest1 to 34.5°C ± 1.7°C at the end of Exercise2. Temperature increased by 2.7°C at the end of Exercise1 compared to the end of Rest1, followed by an insignificant decrease of −0.3°C after Rest 2. After Exercise2, a further increase of 1.5°C occurred compared to Rest2 followed by insignificant cooling of −0.4°C at the end of Rest3. **ii).** Perceived thermal discomfort. Compared with Rest1, PTC across all regions increased for Exercise1 and Exercise2. Across bouts, residual limb PTC was higher than the intact limb. **Secondary: i).** Core temperature change. Findings: Increased by 0.9°C on average during Exercise1 and remained elevated throughout the remainder of the testing session.	0.68
Klute *et al.* 2014; Seattle; 9^24^	Observational study.	TT; 28-73yr	16 thermistor sensors.	**Primary: i)** Skin temperature change. Findings: Initial 30-minute rest: Avg temp 31°C ± 1.5°C. Post-30-minute treadmill walk: Avg temp 34.1°C ± 1.3°C. Final 60minute seated rest: Avg temp 33.2°C ± 1.2°C.	0.64
Williams *et al.* 2018; London; 5^29^	Repeated measure pilot study.	UL TF 3, UL TT 1, BL 1 (R TF, L TT); 19–42yr	12 thermistor sensors. Arduino microcontroller connected to a real-time clock and an SD card.	**Primary: i).** Residual limb skin temperature change. Findings: Phase One - increase after donning the prosthesis. Exercise one: 4 participants showed an increase. 1 participant displayed no notable change. Final rest phase: all participants showed a further increase. **Phase Two:** all participants showed a higher temperature relative to Phase One. However, it was noted that this was unsurprising due to the difference in ambient temperature between the two tests. A maximum change in was observed in participant 5 with an increase of 5.1°C.	0.50

**Abbreviations:** TT: Transtibial amputee, TF: Transfemoral amputee, BL: Bilateral, UL: Unilateral, LLA: Lower-limb amputee, C: Control (person without an amputation), PTC: Perceived thermal comfort, LSR: Local sweat rate, CLASS: Comprehensive Lower Limb Amputee Survey, DAE: Dynamic air exchange, TSB: Total surface-bearing.

### Quality assessment

Following the application of the inclusion and exclusion criteria to the titles and abstracts, all chosen full-text articles were critically appraised using the QualSyst tool. Proposed by the Alberta Heritage Foundation, this tool allows for a quantitative and reproducible method of identifying literature quality by providing an output number.^22^ The assessment of quantitative studies consists of 14 questions. A score of 0 to 2 can be awarded for each question. A summary score is calculated for each paper indicating its quality. The QualSyst tool suggests a cut-off score of 0.75 for a paper to be included in a review article. The summary score calculation process can be seen in **APPENDIX**.

The quality of the paper is then further defined in a literature review with a scoring system defined by Lee et al. as; strong (summary score of >0.80), good (summary score of 0.71–0.79), adequate (summary score of 0.5–0.7) and limited (summary score of <0.5).^23^ The QualSyst score for each study can be seen in the data extraction table (**Table 3**). Due to the low number of included articles, the exclusion of papers lower than the cut-off score was not appropriate, therefore, quality assessment was conducted as an outcome measure to promote discussion, rather than an exclusion criterion.

## RESULTS

**Figure 1:**
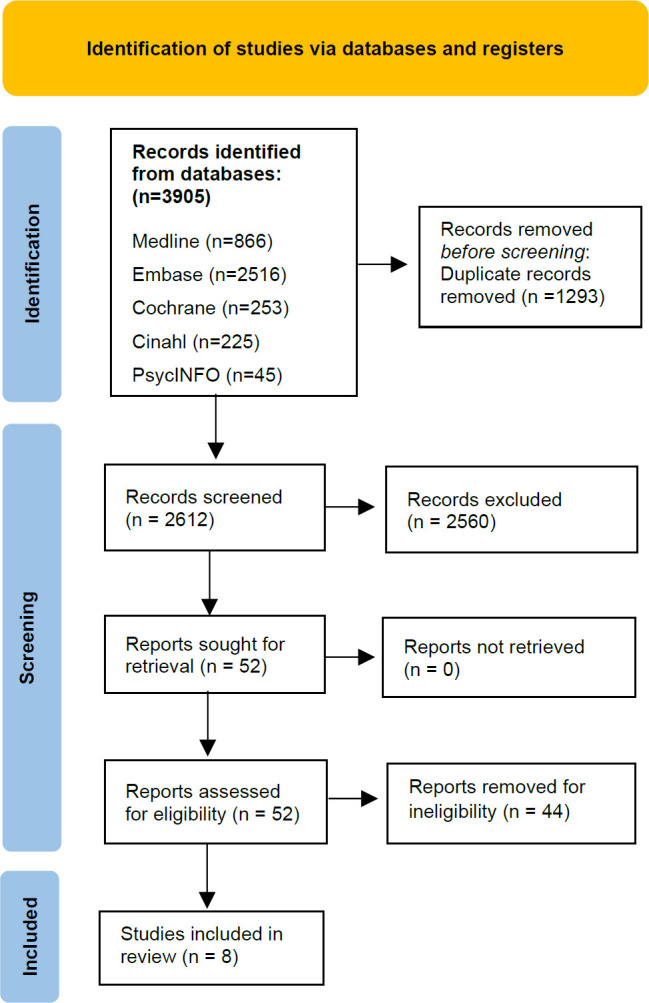
PRISMA flow diagram.

The search yielded 2612 articles after the removal of duplications. After the inclusion and exclusion criteria were applied to the title and abstract of the remaining articles, 8 were taken forward for quality assessment and review. This selection process can be seen in the PRISMA flow chart shown in **Figure 1**. Four studies achieved an adequate score, two achieved a good score and two achieved a strong score. The results discussed in the section are presented in more detail in **Table 3**.

### Countries reported

Five studies reported data from the USA; three from Seattle,^24–26^ one from Ohio,^27^ and one from Indianapolis.^28^ Two studies reported data from the UK; one from London,^29^ and one from Oxford.^5^ One study reported data from Hiroshima, Japan.^30^

### Study method

All eight studies used a quantitative approach. Specifically, four studies used a randomized control trial methodology.^25,27,28,30^ One study used a comparative study methodology,^5^ whilst another used a repeated measure pilot study methodology.^29^ One study used a structured observation study design,^26^ and another utilized an observational experimental methodology.^24^

### Study sample sizes

A total of 96 participants were included across the eight studies, 62 participants with a transtibial amputation from seven studies,^5,24–27,29,30^ 25 participants with a transfemoral amputation from four studies,^5,28–30^ 2 participants with a bilateral amputation from two studies,^27,29^ and 7 participants without an amputation from one study.^30^ One study tested 5 participants with a transfemoral amputation and 2 participants with a transtibial amputation alongside 7 participants without an amputation as a control group for comparison.^30^ Another study involved a participant with a bilateral amputation to conduct a blind test of two interventions simultaneously, allowing for immediate comparison of results.^27^

### Thematic analysis

Within the broad scope of reviewing thermoregulation and thermal discomfort in lower-limb prosthetics, four themes emerged. **Theme 1** – The effect of activity on residual limb skin temperature, and making a comparison to thermal discomfort as a related issue, **Theme 2** Comparing the effect of a prosthetic cooling intervention to a regular non-cooling prosthetic design on thermoregulatory and subjective measures, **Theme 3** – Comparing thermoregulatory and subjective responses between participants with a lower-limb amputation and control participants in a hot environment, **Theme 4** – Measuring residual limb skin temperature to verify out-of-lab thermal comfort studies.

***Theme 1:*** Klute et al. investigated the impact of increasing activity on residual limb skin temperature in lower-limb amputees, commenting on thermal discomfort decreasing quality of life as a result of the increased temperature. The author highlighted the need for developing a cooling intervention to improve thermal comfort and promote prosthetic adherence by reducing local perspiration.^24^ Segal et al. reported an increase in mean residual limb skin temperature and core temperature during exercise testing in a cold environment. Perceived thermal discomfort levels were elevated on the residual limb compared to the contralateral limb post-exercise.^26^ Diment et al. reported both residual and contralateral limbs cooling during exercise and no significant difference in skin temperature between the limbs. Thermal discomfort was greater on the amputated side compared to the contralateral side.^5^

***Theme 2:*** Three studies^25,27,28^ tested a prosthetic cooling intervention against a regular non-cooling prosthesis and reported the differences in residual limb skin temperature and perspiration levels. Two studies compared the thermoregulatory measures against subjective responses including thermal comfort, thermal sensation and prosthetic satisfaction.^25,28^ Klute et al. compared a dynamic air exchange (DAE) prosthetic socket to a total surface-bearing suction socket (Suction). No significant difference in residual limb skin temperature was reported between the groups however a significant reduction in perspiration was observed in the DAE results. Subjective measures revealed better residual limb health and a reduction in heat and sweating when wearing the DAE prosthesis, however, it was found to be more frustrating than the Suction prosthesis.^25^ Gnyawali et al. compared a vented linersocket system (VS, Ossur) with a Seal-in liner and non-vented socket (nVS, Ossur). During activity, relative socket humidity was reduced in the (VS, Ossur) compared to the (nVS, Ossur). However, no significant difference in in-socket temperature between the two systems was reported. There was also no significant difference between subjective measure reports on suspension, comfort or stability between the two systems.^28^ Wernke et al. compared a SmartTemp Phase Change Material liner against a placebo liner and reported a reduction in residual limb skin temperature and local perspiration within the SmartTemp group.^27^

***Theme 3:*** Hasegawa et al. compared both thermoregulatory and subjective responses between participants with lower-limb amputation and a control group of participants without an amputation, under the same exercise conditions in a hot environment. Core temperature increased throughout exercise testing however no difference was observed between the groups. Skin temperature also increased throughout both groups but tended to be higher in the group of participants with a lower limb amputation. Local sweat rate increased for both groups however a difference between the amputated and contralateral limb was observed in the group of participants with a lower-limb amputation, with a higher local sweat rate being observed on the contralateral side. Thermal sensation was lower in the group of participants with a lower limb amputation. Thermal comfort decreased for both groups during exercise however it remained higher within the group of participants with a lower limb amputation.^30^

***Theme 4:*** Williams et al. compared residual limb skin temperature measurements during an exercise test in a controlled environment to measurements obtained during out-of-lab testing of participants engaging in daily activities. Results revealed that on average, residual limb skin temperatures were higher in out-of-lab testing.^29^

## DISCUSSION

To the best of the author’s knowledge, this scoping review represents the first evaluation of literature regarding thermoregulatory measures – such as skin temperature, core temperature or sweating - for addressing thermal discomfort in lower-limb prosthetics. Notably, a previous literature review has been undertaken which explored the prevalence of heat and perspiration discomfort within prosthetics.^3^ The main findings of this review include inconsistent findings on the role of skin temperature in thermal discomfort, raising questions about its significance as a measurable parameter. In addition, thermal discomfort was often linked to sweat accumulation rather than skin temperature changes. Sweat accumulation in the socket may be a major discomfort due to the involvement of wet skin discomfort and mechanoreceptor activation. People with lower-limb amputation may compensate for heat dissipation with increased sweating and blood flow in other body areas.

### Review Scope and Objectives

The findings of this review address the utilization of thermoregulatory measures in testing to investigate thermal-related issues in lower limb prosthetics, to potentially identify novel directions for experimental design in future research.

Despite the importance of addressing thermal discomfort in lower-limb prosthetics and its consequent effect on user satisfaction and quality of life, limited evidence is available at present on how to address the issue. This may be due to a lack of understanding of the effects a prosthesis has on the thermoregulatory processes, how prosthetic liners or sockets interact with the skin and how thermal discomfort is perceived by people with an amputation.

### Skin Temperature Measurements

When addressing the issue of thermal discomfort in lower-limb prosthetics, researchers have measured skin temperature changes at the residual limb as many people with an amputation report elevated skin temperatures and a consequent increase in perspiration to be a cause of discomfort.^3,31–35^ All the studies included in this review measured the change in residual limb skin temperature in response to activity. This may be indicative of residual limb skin temperature being the most widely accepted measurement when carrying out experimental research into lower-limb prosthetic thermal discomfort. Four studies reported a significant increase in residual limb skin temperature during activity.^24,26,29,30^ Both Klute et al. and Wernke et al. reported an overall increase in residual limb skin temperature during activity, however, no significant difference between the DAE Socket and SmartTemp Liner designs was observed compared to the conventional socket and liner.^25,27^ Gynawali et al. reported no significant change in residual skin temperature post-exercise.^28^ Diment et al. reported a slight decrease in residual limb skin temperature on average post-exercise.^5^ The conflict in the results may allude to whether or not residual limb skin temperature may be the prime factor in thermal discomfort.

### Perspiration Measurements

As a reported consequence of an increased thermal environment at the BDI, lower-limb prosthetic users also commonly report increased perspiration within the socket leading to sweat build-up. This has directed research towards improving socket and liner designs to reduce perspiration levels.

Four of the eight included studies measured perspiration within the socket during testing. Klute et al., Wernke et al. and Gnyawali et al. all reported an increase in sweat or humidity at the BDI during exercise.^25,27,28^ Wernke et al. also disclosed that 4 of the 16 subjects did not sweat in either of the tested liners during exercise.^27^ Hasegawa et al. reported an increase in local sweat rate at the thigh, with the prosthesis removed for the group of participants with an amputation.^30^

### Prosthetic Interventions for Thermal-Related Issues

Prosthetic technologies have been engineered to mitigate heat and perspiration, serving as a targeted intervention. Such interventions include sockets which incorporate cooling mechanisms such as heat pumps or cooling channels and liners composed of phase change materials which can store and release heat, as presented in **Table 3**.^2,27,36^ Klute et al., Wernke et al. and Gnyawali et al. conducted studies assessing the efficacy of an intervention device in contrast to conventional prosthetic components. Their research aimed to elucidate the effects of these interventions on thermoregulatory parameters during exercise.^25,27,28^ Klute et al. and Gynawali et al. both compared a conventional socket to an intervention socket, designed to expel accumulated perspiration and discussed changes in residual limb skin temperature, sweating and subjective measures. Wernke et al. compared a placebo liner to a SmartTemp liner composed of phase change material.^27^ As previously discussed, all three studies reported a significant reduction in perspiration levels during activity. However, no significant change in skin temperature was recorded. Participants also rated perceived limb perspiration to be lower when using the intervention sockets compared to the standard sockets.^25,28^ The outcomes from these studies suggest that prosthetic interventions targeting heat and perspiration reduction were significantly more proficient in lowering perspiration levels compared to their impact on residual limb temperature.

### Impact of Amputation on Heat Dissipation

The process of amputation, by nature, reduces the surface area of the human body. Body surface area is reduced by approximately 21% for a transfemoral amputation and 9% for a transtibial amputation.^35^ This may impair a person with an amputation's ability to dissipate heat as there is less area for convection, radiation, evaporation and conduction to occur.^3^ Hasegawa et al. measured local sweat rate, skin blood flow at the thigh, and total sweat rate in both participants with an amputation and control participants without an amputation. In the control group, both skin blood flow and local sweat rate increased symmetrically across both limbs during exercise. In the group of participants with an amputation, skin blood flow measurements were significantly higher in the contralateral limb compared to the residual limb during exercise. Similarly, local sweat rate was significantly higher on the contralateral limb compared to the residual limb. Despite these differences, the total amount of local sweat rate across both limbs did not differ between the two groups. However, the total sweat rate was significantly higher in the group of participants with an amputation. Hasegawa et al. suggested that despite the lower body surface area for heat dissipation in people with an amputation, the overall heat dissipation was similar between the two groups. This was likely due to compensatory sweating on the torso and contralateral limb. They also inferred that increased blood flow on the contralateral side might indicate compensatory vasodilation on the limb with an amputation.^30^ This was supported by Diment et al. who found that the contralateral limb was typically warmer than the residual limb, even with the prosthesis donned when measuring skin temperature during exercise.^5^ The consistency in these findings indicates a compensatory role of heat dissipation following amputation.^30^

### Subjective Measures for Thermal Discomfort

Both socket and thermal comfort and discomfort are inherently subjective, difficult to distinguish and vary from individual to individual, posing challenges to reproducibility and reliability as a measurable parameter.^37^ However, within the domain of prosthetics, it holds significant importance for researchers as it aids the identification of specific scenarios or environmental conditions associated with increased thermal discomfort. Thermal comfort and discomfort are commonly assessed through subjective measure questionnaires, whereby users report on comfort, heat levels, perspiration, suspension and functionality of their prosthesis. Five of the included studies recorded subjective measures.^5,25,26,28,30^ Segal et al., Diment et al. and Hasegawa et al. all measured thermal comfort or discomfort as a direct outcome.^5,26,30^ All three studies agreed that thermal discomfort increased at the residual limb with exercise. When comparing thermal discomfort between the residual and contralateral limb, both Segal et al. and Diment et al. reported heightened thermal discomfort at the residual limb compared to the contralateral limb.^5,26^ Parallel to this, when comparing a group of participants with an amputation to a control group without an amputation, Hasegawa et al. reported an increase in thermal discomfort during exercise across all limbs in both groups. Interestingly, thermal discomfort was significantly lower on average in the group of participants with an amputation than in the control group. This may, however, be a result of testing being conducted with the prosthesis removed, suggesting people with an amputation may be more tolerant to thermal discomfort as the issue is experienced much more often compared to a person without an amputation.^30^

### Correlation Between Thermoregulatory Parameters and Subjective Measures

To gain a better understanding as to what may be causing thermal discomfort for people with a lower limb amputation, it is important to draw links between the subjective responses and thermoregulatory measurements gathered during testing. Klute et al. discussed a general increase in residual limb skin temperature for both intervention and conventional sockets tested, although, no significant difference was seen between the two during exercise. The intervention socket significantly reduced the amount of accumulated sweat compared to the conventional socket. Subjects reported that the intervention socket reduced heat and sweat. They also “strongly agreed” with the statement that the intervention socket kept their residual limb at a more comfortable temperature whereas they “slightly disagreed” when wearing the standard socket.^25^ Segal et al. reported a significant increase in skin temperature during exercise, coinciding with an increase in thermal discomfort. Thermal discomfort tended to be higher at the residual limb compared to the contralateral limb.^26^ Hasegawa et al. reported an increase in skin temperature post-exercise in both groups of participants with an amputation and control groups along with an increase in local sweat rate and thermal discomfort.^30^ Gnyawali et al. reported no significant increase in residual limb skin temperature for both intervention and conventional sockets throughout testing. A significant increase in residual limb humidity measurements was observed during exercise. The perceived sweat score was reported significantly lower for subjects wearing the intervention compared to the conventional socket, however, The Comprehensive Lower Limb Amputee Survey (CLASS), used when assessing prosthetic fit, showed no significant difference between the groups for suspension, comfort or stability.^28^ Diment et al reported a decrease in residual limb skin temperature during exercise, however, thermal discomfort was still experienced, more so on the amputated side.^5^

An intriguing link between thermoregulatory parameters and subjective measures was made. Both Klute et al. and Gnyawali et al. reported a decrease in thermal discomfort when subjects were wearing a socket designed to reduce heat and perspiration, however, participants could not be blinded to the intervention sockets. Both interventions also reduced the amount of accumulated sweat within the socket. However, Klute et al. reported no significant difference in the increase of residual limb temperature between the two sockets. Gynawali et al. also reported the lack of a significant increase in skin temperature for both sockets.^25,28^

Considering the reduction in thermal discomfort, coinciding with a reduction in accumulated sweat, without any significant changes in residual limb skin temperature, suggests that skin temperature may not be the primary instigator for lower-limb thermal discomfort. Furthermore, Diment et al. concluded that skin temperature does not explain the thermal discomfort experienced by the prosthetic users in their study.^5^ However, it is noteworthy that both Hasegawa et al. and Segal et al. reported an elevation in thermal discomfort alongside an increase in residual limb skin temperature.^26,30^

### Behavioral Thermoregulation

Shlader et al. explored behavioral thermoregulation as the most favored and effective form of temperature regulation. They demonstrated that eliciting this behavior does not require a temperature change; rather, thermal sensation and discomfort alone suffice as triggers.^38^ Discussing the findings of Diment et al., even in the absence of a rise in residual limb skin temperature, the presence of thermal discomfort alone during exercise may prompt the person with an amputation to remove their prosthesis as a behavioral thermoregulatory response.^5^

### Future Research Directions

These findings pose the question of how thermal discomfort is perceived by a person with an amputation when wearing a prosthesis. An avenue for future research may be to explore the effects of prolonged skin wetness due to sweat accumulation at the BDI. Detecting skin wetness has been shown to impact thermal comfort, and therefore thermoregulatory behavior which makes it a crucial mechanism for thermal adaptation.^39^ Humans detect skin wetness through multisensory integration between thermal and mechanosensory inputs.^40^ Under normal thermoregulatory circumstances, temperature sensation and thermal discomfort are linked to skin surface temperature in cold environments. In warm environments, on the other hand, thermal discomfort is more related to sweating than skin temperature, as skin temperature is maintained at a favorable level due to evaporative cooling due to sweating. It is suggested that one factor for this thermal discomfort is the level of wetness over the skin surface.^41^

### Limitations

A limitation of this study was the inclusion of low-quality articles due to the low number of included literature. As aforementioned, only two articles achieved a strong QualSyst score of >0.80, two articles achieved a good QualSyst score of 0.71-0.79 and four articles achieved an adequate QualSyst score of 0.5-0.7. Referring to the QualSyst scores in the extraction table, five of the included articles scored below the suggested QualSyst cut-off score of 0.75, and if the score was used as an exclusion criterion instead of an outcome measure as per this review, these articles would have been removed.

## CONCLUSION

This scoping review evaluated the current literature concerning thermoregulatory measures associated with thermal discomfort in lower-limb prosthetics. Whilst literature has explored the prevalence of heat and perspiration discomfort within prosthetics, this review investigated the utilization of thermoregulatory measures for addressing thermal-related issues.

Although numerous studies have tested thermal discomfort in lower-limb prosthetics, there may be a lack of understanding of how donning a prosthesis affects thermoregulation from a physiological point of view. Measuring the change in residual limb skin temperature has been the focal point for discussing thermal discomfort in lower limb prosthetics, with several studies reporting elevated skin temperatures during exercise. However, conflicting results reporting a reduction or no change in skin temperature pose questions about the significance of the parameter as the prime factor in lower-limb prosthetic thermal discomfort.

Another significant aspect contributing to prosthetic thermal discomfort is perspiration at the BDI leading to the accumulation of unevaporated sweat. Prosthetic interventions designed to reduce heat and perspiration at the BDI show promise for reducing perspiration and sweat accumulation, consequently improving thermal comfort, emphasizing the importance of technological advancements in this area. However, little evidence emerged regarding the efficacy of these interventions for reducing residual limb skin temperature, with a lack of correlation between skin temperature measurements and thermal discomfort at the BDI.

Whilst progress has been made to combat and further understand lower limb prosthetic thermal discomfort, both through design and research, a knowledge gap remains. In particular, regarding the perception of thermal discomfort and the involvement of not only skin temperature but also the effects of prolonged skin wetness due to unevaporated sweat. This may be a pivotal parameter for further managing thermal discomfort, increasing usage and improving the quality of life for lower-limb prosthetic users.

## APPENDIX

Alberta Foundation QualSyst tool.

**Table:**

Criter		YES (2)	PARTIAL(1)	NO (0)	N/A
1	Question/objective sufficiently described?				
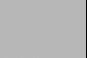
2	Study design evident and appropriate?				
3	Method of subject/comparison group selection or source of information/input variables described and appropriate?				
4	Method of subject/comparison group selection or source of information/input variables described and appropriate?				
5	If interventional and random allocation was possible, was it described?				
6	If interventional and blinding of investigators was possible, was it reported?				
7	If interventional and blinding of subjects was possible, was it reported?				
8	Outcome and (if applicable) exposure measure(s) well defined and robust to measurement/misclassification bias? Means of assessment reported?				
9	Sample size appropriate?				
10	Analytic methods described/justified and appropriate?				
11	Some estimate of variance is reported for the main results?				
12	Controlled for confounding?				
13	Results reported in sufficient detail?				
14	Conclusions supported by the results?				

The grey highlights indicate that the ‘N/A’ box cannot be checked for that criteria question. A summary score is calculated for each paper by summing the total score obtained across relevant items and dividing by the total possible score (i.e.: 28 – (number of “n/a” x2)).^22^
